# Prospective Study on the Impact of Fear of Falling on Functional Decline among Community Dwelling Elderly Women

**DOI:** 10.3390/ijerph14050469

**Published:** 2017-04-27

**Authors:** Kyungwon Choi, Gyeong-Suk Jeon, Sung-il Cho

**Affiliations:** 1 Department of Nursing, Korea National University of Transportation, 61 Daehak-ro, Jeungpyeong-gun Chungbuk 27909, Korea; kwchoi@ut.ac.kr; 2 Department of Nursing, Mokpo National University, 1666 Yeongsan-ro, Cheonggye-myeon, Muan-gun, Jeollanam-do 534-729, Korea; 3 Graduate School of Public Health and Institute of Health and Environment, Seoul National University, 1 Gwanak-ro, Gwanak-gu, Seoul 08826, Korea; scho@snu.ac.kr

**Keywords:** fear of falling, functional decline, elderly, disability

## Abstract

Fear of falling (FOF) is expected to have effects on functional decline in the elderly. In this study, we examined over 2 years the effect of change in FOF on functional decline in community dwelling elderly. We conducted a secondary analysis using data from elderly women, 70 years of age and older, who participated in the Korean Longitudinal Study of Aging (KLoSA). Participants were divided into four categories according to change in FOF between the 2010 and 2012 surveys. Multiple logistic regression analysis was conducted regarding the effects of changes in FOF on functional decline after controlling for variables as known risk factors for functional decline. Rates of functional decline were highest in the “consistently having FOF” group, whereas they were lowest in the “consistently no FOF” group in both 2010 and 2012. Characteristics independently associated with functional decline were change in FOF, depressive symptoms, low frequency of meeting friends, and fear-induced activity avoidance. Longer exposure to FOF was associated with an increased risk of functional decline. FOF is an important health problem that deserves attention in its own right. Public health approaches for elderly persons should address early detection, prevention, and intervention programs for FOF.

## 1. Introduction

Fear of falling (FOF) has long been considered to be a result of psychological distress after a fall, also called “post-fall syndrome” [1], and has been identified as a potentially modifiable threat to the autonomy and quality of life in older adults [2,3]. However, FOF is also prevalent in older adults who have not yet experienced a fall [4]. Regardless of any history of falls, the prevalence of FOF is reported to range between 20% and 85% among community dwelling older adults [4,5,6], with women being affected significantly more [3,4].

FOF is considered to contribute to a loss of independence or disability through the avoidance and restriction of activities [2,7,8]. However, not all elderly with FOF avoid activities in daily life. FOF has a range of consequences, from increased caution during the performance of daily activities, which may be protective against falls, to a complete restriction of activities, which may be devastating [9]. FOF, unless it induces excessive activity avoidance, may indeed be helpful, because it increases caution in daily activities [6]. Fear-induced activity avoidance, when present at an excessive level, may actually lead to a deconditioning effect on muscle strength and postural control, which may further contribute to needless restrictions in participation in physical and social activities, resulting in further muscle atrophy, lower mobility, and worse balance, increasing the risk of falling, social isolation, depression, and functional decline [7,10,11]. Thus, the main problem in FOF’s negative consequences seems to be fear-induced activity avoidance and the resulting physical weakness. In fact, fear-induced activity avoidance has been reported to predict deterioration in physical functioning and a decreased level of activity in prospective study [10,12].

Meanwhile, recent several prospective studies have shown the independent contribution of FOF on functional decline, and suggested that FOF is a long-lasting condition associated with declines in physical function [2,7,8]. However, these studies were not considered the possible mediating factors such as activity avoidance, social interaction, and depression between FOF and physical function. For example, several researchers reported that FOF reduces social contacts with friends and family [3,13], which supported the notion that FOF may have the constraining effects on social contact [3]. Murphy et al. [5], however, reported that lack of social interaction was not associated with fear-induced activity avoidance. Depressive symptoms, another important risk factor for functional decline [14], were significantly associated with FOF, but not with activity restriction in Vand an Haastregt et al.’s study [15]. While, Murphy et al. [5] reported that depression was a factor that distinguished those with fear-induced activity avoidance form those with fear of falling alone. Based on previous research, it is likely that FOF may lead to activity avoidance or decreased social interaction, which then contribute to depression in the elderly [16,17]. However, there is no general consensus with regard to the relationships among FOF and induced activity avoidance, social interaction, and depressive symptoms, and exists a paucity in understanding the direct effect of FOF to functional decline. This gap creates a need to evaluate the net effect of FOF with controlling for the mediating effect of these factors between FOF and functional decline.

The aims of this study were to examine prospectively the relationship between FOF and functional decline and to examine its extent after controlling for the influence of the potential mediators such as fear-induced activity avoidance, lack of social interaction, and depressive symptoms among community dwelling elderly women. Specifically, we hypothesized that the presence of FOF would have a direct effect on functional decline, and longer duration of FOF would increase the risk of functional decline.

## 2. Methods

### 2.1. Data Collection and Study Participants

This study used a sample from the third and fourth wave data of the Korean Longitudinal Study of Aging (KLoSA). This study is being conducted by the Korea Labor Institute to create a basic data set needed to devise and implement effective social and economic policies that address emerging trends related to the aging of the population. KLoSA is an ongoing longitudinal panel survey of a nationally representative sample of community dwelling adults aged 45 years and older who were alive and lived in 15 large administrative areas at the time of the baseline interview in 2006. The survey has been repeated every even-numbered year, and is based on a multistage stratified area probability sample of households representing the entire population of South Korea. Before the data collection, all participants provided written informed consent. Detailed information on the sampling design and survey approach can be found at the KLoSA website (http://www.klosa.re.kr).

In the present study, we restricted our population to elderly women who were 70 years old in 2010 because the majority of elderly men did not report FOF. Of these 70 older females participants in Wave 3 (*n* = 1819), 1600 completed the Wave 4 interviews in 2012. Attrition between Wave 3 and Wave 4 was caused by refusal, failure to contact, and mortality (219 were lost to follow-up). We also excluded participants with missing data (*n* = 40), leaving a final sample of 1560 elderly women.

### 2.2. Measurements

To assess the net effect of changes related to FOF in functional decline, the three variables “fear-induced activity avoidance”, “frequency of meeting friends”, and “depressive symptoms” were employed as explanatory variables. These variables were considered to potential mediators which might be consequences of FOF in previous studies [3,5,10,18,19]. These data come from the 2012 survey. Meanwhile, number of chronic disease, cognitive function, sensory function, and experience of falling in 2010 were included as covariates because these were considered to confounding factors of FOF and functional decline [14].

#### 2.2.1. Functional Decline

Functional decline is usually defined as a decrease in functional status to perform activities of daily living items [20]. In this study, functional decline was defined as having disability in 2012 adjusted by disability in 2010. Functional status of both 2010 and 2012 was measured using the Korean versions of the Activities of Daily Living (ADL) and Instrumental Activities of Daily Living (IADL) scales. Respondents were asked whether they needed assistance when performing seven daily activities (dressing, washing face/shampooing/teeth-brushing, bathing, eating meals, getting up and moving out of the room, using the toilet, and controlling urination and defecation) and 10 different instrumental activities (personal grooming, doing house chores, preparing meals, doing the laundry, going outside without using transportation, going outside using transportation, shopping, managing money, using the telephone, and taking medications) [21]. If the participants needed someone’s help for one or more activities, they were categorized as having disability.

#### 2.2.2. Fear of Falling (FOF)

FOF was assessed by the question “Are you afraid of falling?” Possible answers to the question were not at all, slightly, and very much. Participants who were not fearful of falling or reported slight FOF were classified as “no”; participants who reported that they were fearful of falling very much were classified as “yes”. Participants were divided into four categories according to changes in FOF between the 2010 and 2012 surveys: no FOF in both 2010 and 2012 (*n* = 761), having FOF in 2010 and no FOF in 2012 (*n* = 199), no FOF in 2010 and having FOF in 2012 (*n* = 243), and having FOF in both 2010 and 2012 (*n* = 357).

#### 2.2.3. Depressive Symptoms

Depressive symptoms were evaluated using the short-form Center for Epidemiological Studies (CES-D10), which consists of 10 items and assesses the type of symptoms experienced during the most recent week [22]. Eight items were negatively stated, and two items were positively stated. The scores for each item ranged from 0 (very rarely; less than 1 day during the past week) to 3 (almost always; 5–7 days per week). The summed scores of the 10 items, with scores reversed for the positively stated items, were calculated. We used the cut-off score of ≥10 for the CES-D10 to identify older adults with symptoms of clinical depression.

#### 2.2.4. Fear-Induced Activity Avoidance

Fear-induced activity avoidance was assessed by the question “Have you had any activities or types of work that you wanted to do, but did not try or avoided, because you were afraid of falling?” (yes or no).

#### 2.2.5. Frequency of Meeting Friends

The frequency of meeting friends was measured based on the question, “Do you have close friends, relatives, or neighborhood friends? If you have, how often do you get together with them?” The participants were asked to respond on a 10-point Likert-type scale (1 = none, 2 = rarely, 9 = 2–3 times a week, 10 = every day). Responses were grouped into three: less than several times a year or none, once or twice a month, and more than once a week.

#### 2.2.6. Covariates

To assess the independent effect of FOF in functional decline, we considered the possible predictors of fear of falling or functional decline identified by the results of previous studies, including age, marital status and living arrangement (no spouse and living alone/no spouse and living with/living with others/living with spouse and others), education (elementary school or less, middle school, high school or more), experiences of falling, number of chronic disease, cognitive function, sensory function (vision, hearing), and disability in 2010 as covariates. With regard to experiences of falling, participants were asked whether they had fallen during the past 2 years (yes or no). A fall was defined as an unintentional change in body position resulting in contact with the ground or with another lower level [23]. Number of chronic diseases was measured by self-reported disease history. Participants reported one or more physician-diagnosed diseases, including hypertension, diabetes, cancer, lung disease, hepatic disease, cardiac disorders, stroke, and arthritis. Cognitive function was assessed using the Korean version of the Mini-Mental State Examination (K-MMSE) [24], which is widely used for screening of cognitive impairment among older adults. Total score range from 0 to 30, with higher scores indicating better cognitive function. Validity and reliability of K-MMSE instrument were established [24]. K-MMSE scores were categorized as severe cognitive impairment (K-MMSE ≤ 17), mild cognitive impairment (18 ≤ K-MMSE ≤ 23), and normal cognitive function (K-MMSE ≥ 24), by the conventional classification criteria for cognitive function [24]. Visual and auditory functions were assessed using a single-item measure with a five-point rating scale. To evaluate visual impairment, the participants were asked to rate their eyesight using the question “How is your eyesight when wearing your glasses or contact lenses?” Auditory impairment was assessed with the question “How is your hearing when wearing your hearing aid?” Responses were classified as “good” (very good or good) or “poor” (fair, bad or very bad).

### 2.3. Statistical Analysis

We present frequencies and means (±standard deviation (SD)) of the baseline characteristics of the participants and compared them with the four categories using χ^2^ tests and ANOVA. Multiple logistic regression analysis was used to evaluate the influence of change in FOF over 2 years on functional decline. The model was adjusted for age, marital status and living arrangement, education, experience of falling, number of chronic disease, cognitive function, sensory function (vision, hearing), and disability in 2010: age was entered into the model as a continuous variable, and all other covariates were entered as categorical variables. Statistical analyses were carried out with the SPSS software (v. 22.0 for Windows; SPSS, Inc., Chicago, IL, USA).

## 3. Results

Table 1 shows the sociodemographic and health characteristics of the study population according to change in FOF. Almost half of the participants (48.8%) were included in the “consistently no FOF” group, 22.9% were consistently afraid of falling during the 2 years, and 28.4% experienced changes in FOF. The mean age across all groups was 77.17 years, and the “consistently having FOF” group was the oldest. “Consistently having FOF” group members were more likely to have no spouse, and to have more experience of falling, more impaired cognitive function, worse sensory function, and depressive symptoms than those in other groups. The “consistently having FOF” group also reported the largest number of chronic diseases, whereas the “consistently no FOF” group had the fewest. Rates of fear-induced activity avoidance were overwhelmingly higher in the “consistently having FOF” and “no FOF—having FOF” groups.

Table 2 shows the prevalence of disability in 2012 by the sociodemographic and health characteristics across four groups. About twenty one percent of Korean older women aged 70 years or older have showed disability in 2012. Significant differences in prevalence of disability of 2012 were found among four groups by different fear of falling status. “Consistently having FOF” group (49.0%) showed the highest prevalence of disability in 2012, whereas “consistently no FOF” group (7.1%) showed lowest. Moreover, in all categories of the variables, “consistently having FOF” group were more likely to have disability than other groups were. Significant differences in prevalence of disability of 2012 were found among four groups with the same levels of fear-induced activity avoidance and depressive symptoms. Although it was not statistically significant across the groups, older Korean women who meet friends less than several times a year or none showed more disability in 2012 than those who do not. Among covariates, disability in 2010, impaired cognitive function, and bad visual function were each significantly associated with a higher prevalence of disability in 2012.

Table 3 presents the results of the multivariate logistic regression analysis, as odds ratio (ORs) and 95% confidence intervals (CIs), assessing the association between fear of falling and functional decline, along with the compounding and mediating factors. Model 1 included change in FOF and compounding factors (age, education, marital status and living arrangement, fall history, number of chronic disease, cognitive function, sensory function and disability in 2010) only as a baseline model. The “having had FOF-no FOF,” “no FOF-having had FOF,” and “consistently having FOF” groups had higher likelihoods of functional decline compared with the “consistently no FOF” group as a reference; the corresponding ORs (95% CIs) were 1.90 (1.14–3.19), 3.74 (2.36–5.94), and 6.01 (3.97–9.11), respectively. In Models 2–4, health characteristics regarded as mediating factors for functional decline were added to Model 1 in sequence. Each variable decreased the odds of change in FOF for functional decline, and the reduction of odds showed similar patterns of associations to those in Model 1. The magnitude of the association between change in FOF and functional decline decreased slightly but still remained significant in Model 5, in which all the possible mediating factors were simultaneously entered. The “no FOF-having had FOF” and “consistently having FOF” groups had twice (OR = 2.26, 95% CI = 1.36–3.75) and three times (OR = 3.43, 95% CI = 2.15–5.47) higher risk of functional decline than their reference group, respectively. In addition, activity avoidance, low frequency of meeting friends (less than several times a year or none), and depressive symptoms were independently associated with functional decline.

## 4. Discussion

In this unique prospective study, we measured the change in FOF over time and observed the independent impact of FOF on functional decline in community dwelling elderly women. Importantly, we found that longer duration of FOF was associated with an increased risk of functional decline, and its’ effect was much stronger than other risk factors’ mediating effect. These findings support the idea that FOF is likely to be a causal factor of poor physical function in older people.

FOF has been considered to be a risk factor because it leads to avoidance of activities and, thus, to physical deconditioning [25,26]. Our findings suggest that this may not be the case, and support the notion that FOF is an important health problem that deserves attention in its own right. This is not to say that avoidance of activities has no role in functional decline. As shown in the results, fear-induced activity avoidance partially mediated the effect of FOF on functional decline. However, at least with community dwelling elderly, avoidance of activities seems to be far less important in predicting functional decline than FOF itself. This result supports Hadjistavropoulos et al.’s conceptual framework that FOF predicts future falls in community dwelling older persons, not avoidance of activities [10]. Furthermore, we found that elderly persons with persistent FOF over the two surveys showed a higher risk of functional decline (OR = 3.43, 95% CI = 2.15–5.47) than did those with a recent, 2-year exposure, to FOF (OR = 2.26, 95% CI = 1.36–3.75), and those having had FOF in the past (OR = 1.60, 95% 0.95–2.72). The clear dose-response between FOF and functional decline shown in this study adds support to this previous prospective study [10]. Therefore, FOF itself may make a distinct contribution to functioning in older persons. So, how does FOF itself, not through fear-induced activity avoidance, contribute to functional decline? We propose that it might be due to FOF contributing to an increased risk of falls. According to psychological theory, increased anxiety could cause attentional bias towards a threat and compromise the efficiency of working memory necessary to execute complex locomotor tasks [27]. For example, older adults with FOF showed an incongruent response to fall-threat words and this caused them to have some difficulty in disengaging from fall-threatening stimuli [28]. Moreover, gaze diversion, due to anxiety, in older adults with FOF may increase walking instability [29]. A recent study showed that older adults with higher FOF demonstrated earlier, longer, and more frequent fixation on an initial target than a subsequent target, which caused frequent missed steps. Older adults without FOF, however, frequently transferred visual fixation between obstacles and made fewer step errors during their travel path [30]. Several studies have also shown that FOF is associated with premature gaze transfer away from a target, compromising safe gait that could lead to an increased fall risk in older adults [31,32]. Our current discussion on the relationship between FOF and the possibility of functional decline, based on linkage of changes in attentional processes and visual diversion, is still limited due to lack of previous research. Thus, further research is needed to establish basic causal links between FOF and altered attentional processes and functional decline in older people.

The association of functional decline with avoidance of activities and psychosocial factors in both univariate and multivariate analyses agrees with the results of previous studies [3,5,7,33]. Interestingly, not all elderly with FOF showed an avoidance of activities, and some elderly who were not afraid of falling did report an avoidance of activities. In accordance with our findings, Howland et al. reported that avoidance of activities was not just associated with extreme levels of fear, and proposed that older persons may feel safer by coping with their fear effectively through avoiding activities [3]. According to our further stratified analyses not shown in the tables, those who were not afraid of falling, but avoided activities, actually had the most frequent meetings with friends.

Regarding the frequency of meeting friends, this finding has something in common with the report of Howland et al. that those who could rely on others or talk with friends about falling were less likely to report fear-induced activity avoidance, and social support may be an important prerequisite for continuing to remain active even in the face of FOF [3]. Social support mediates the impact of negative and stressful events on physical and emotional health [34]. This protective effect of social support may differ across the structural and functional characteristics of social relationships (such as social network size, number of social interactions, and amount of instrumental and emotional support) and health status in the elderly [35,36], and these associations may be more complex than simple causal effects. However, our result that structural aspects of social relationships reduced the risk of functional decline in elderly women, when adjusting for other important risk factors, supports the findings that informal social activity with friends was significantly related to decreased disability and mortality risk in later life [36,37].

The relationships between FOF and depression and between depression and functional decline are likely to be bi-directional [9,16,17,38]. Depression is a consequence of FOF as well as risk factor contributing to FOF [4,5], and has been reported to have strong effects on daily functioning [39]. In contrast, disability has also been found to predict the onset and persistence of depression [40,41]. Consistent with these previous reports, the present results suggest that FOF accompanying depressive symptoms accelerates functional decline in an older population. This means that interventions designed to prevent functional decline should include programs for reducing both FOF and depressive symptoms.

Some limitations of the present study should be acknowledged. First, our study was limited with respect to the single-item measures used to assess FOF. Although the single-item assessments may have had limited reliability and present difficulties in direct comparisons with studies that used more sophisticated measures to assess FOF, we nevertheless demonstrated that FOF was associated with known predictors in the expected direction. Additionally, several other studies have used single-item measures successfully to assess FOF [14,25,26]. Another limitation was that we measured fear-induced activity avoidance in a general way, and not in relation to specific activities, such as the basic activities of daily living. Finally, our study did not include tests of physical performance, which may have had significant associations with both FOF and functional decline, because these were not included in the KLoSA data set. Further studies should include objective measures of balance, gait, and muscle strength to establish a more comprehensive understanding.

## 5. Conclusions

This study examined the association between FOF and functional decline in the context of changes in physical capacity and FOF that occurred in the population with longitudinal data on functional status as well FOF. We suggest that FOF itself has a significant role in functional decline in older persons, even after adjusting for risk factors for functional decline including fear-induced activity avoidance, lack of social interaction, and depressive symptoms. The results of this study provide further impetus for the notion that public health approaches for older persons should include early detection, prevention, and intervention programs for FOF.

## Figures and Tables

**Table 1:** Distribution of socio-demographic and health related characteristics by different fear of falling status among Korean older women aged 70 years or older.

	All *n* (%)	No FOF in 2012		Having FOF in 2012	
		No FOF in 2010 *n* (%)	Having FOF in 2010 *n* (%)	No FOF in 2010 *n* (%)	Having FOF in 2010 *n* (%)
	1560 (100.0)	761 (48.8)	199 (12.8)	243 (15.6)	357 (22.9)
Age, years (mean ± SD) *	77.17 ± 5.64	75.47 ± 4.69	77.89 ± 5.55	77.47 ± 5.73	80.20 ± 6.08
Marital status and living arrangement *					
No spouse & Living alone	504 (32.3)	227 (29.8)	65 (32.7)	92 (37.9)	120 (33.6)
No spouse & living with others	445 (28.5)	184 (24.2)	61 (30.7)	62 (25.5)	138 (38.7)
Living with spouse only	444 (28.5)	257 (33.8)	48 (24.1)	66 (27.2)	73 (20.7)
Living with spouse & others	167 (10.7)	93 (12.2)	25 (12.6)	23 (9.5)	26 (7.3)
Education *					
Elementary school or less	1353 (86.7)	631 (82.9)	183 (92.0)	209 (86.0)	331 (92.4)
Middle school	108 (6.9)	65 (8.5)	9 (4.5)	20 (8.2)	14 (3.9)
High school or more	99 (6.4)	65 (7.0)	7 (3.0)	14 (4.5)	12 (3.4)
Having disability in 2010 *					
No	1271 (81.5)	696 (91.5)	153 (76.9)	204 (84.0)	218 (61.1)
Yes	289 (18.5)	65 (8.5)	46 (23.1)	39 (16.0)	139 (38.9)
Having fall history in 2010 *					
No	1489 (95.4)	752 (98.8)	184 (92.5)	236 (97.1)	317 (88.8)
Yes	71 (4.6)	9 (1.2)	15 (7.5)	7 (2.9)	40 (11.2)
Number of chronic disease in 2010 *					
None	1310 (84.0)	668 (87.8)	168 (84.4)	191 (78.6)	283 (79.3)
One or more	250 (16.0)	93 (12.2)	31 (15.6)	52 (21.4)	74 (20.7)
Cognitive function in 2010					
Normal (K-MMSE ≥ 24)	488 (31.3)	324 (42.6)	35 (17.6)	73 (30.0)	56 (15.7)
Mild impairment (18 ≤ K-MMSE ≤ 23)	544 (34.9)	253 (33.2)	80 (40.2)	96 (39.5)	115 (32.2)
Severe impairment (K-MMSE ≤ 17)	528 (33.8)	184 (24.2)	84 (42.2)	74 (30.5)	186 (52.1)
Visual function of 2010 in 2010 *					
Good	944 (60.5)	543 (71.4)	99 (49.7)	144 (59.3)	158 (44.3)
Bad	616 (39.5)	218 (28.6)	100 (50.3)	99 (40.7)	199 (55.8)
Auditory function in 2010 *					
Good	1332 (92.9)	707 (92.9)	156 (78.4)	202 (83.1)	267 (74.8)
Bad	228 (14.6)	54 (7.1)	43 (21.6)	41 (16.9)	90 (25.2)
Fear-induced activity avoidance *					
No	883 (56.6)	602 (79.1)	143 (71.9)	69 (28.4)	69 (19.3)
Yes	677 (43.4)	159 (20.9)	56 (28.1)	174 (71.6)	288 (80.7)
Frequency of meeting friends *					
Less than several times a year or none	261 (16.7)	77 (10.1)	44 (22.1)	42 (17.3)	98 (27.5)
Once or twice a month	179 (11.5)	104 (13.7)	19 (9.5)	23 (9.5)	33 (9.2)
Everyday	1120 (71.8)	580 (76.2)	136 (68.3)	178 (73.3)	226 (63.3)
Depressive symptoms *					
No	951 (61.0)	565 (74.2)	116 (58.3)	126 (51.9)	144 (40.3)
Yes (CES-D10 score ≥ 10)	609 (39.0)	196 (25.8)	83 (41.7)	117 (48.1)	213 (59.7)

* *p* < 0.001 for difference among four groups by different fear of falling status; Notes: SD = standard deviation.

**Table 2:** The prevalence of disability in 2012 according to socio-demographic and health related characteristics among Korean older women aged 70 years or older.

	Disability in 2012 (%)				
	All *n* (%)	No FOF in 2012		Having FOF in 2012	
		No FOF in 2010	Having FOF in 2010	No FOF in 2010	Having FOF in 2010
**Prevalence of Disability in 2012** ^††^	**21.2**	**7.1**	**21.1**	**24.7**	**49.0**
Age group		**	*	**	**
70–74	7.6	2.9	8.6	8.9	28.9
75–79	18.3	6.9	27.8	23.4	38.4
80–84	32.9	14.3	19.1	36.2	53.8
85 and over	56.0	30.0	42.9	58.6	73.1
Marital status and living arrangement	**	*	*		**
No spouse & Living alone	17.1	4.8	9.2	26.1	37.5
No spouse & living with others	35.1	13.6	37.7	33.9	63.0
Living with spouse only	14.0	4.3	18.8	16.7	42.5
Living with spouse & others	16.2	7.5	16.0	17.4	46.2
Education	**				
Elementary school or less	22.8	7.8	21.7	25.9	50.5
Middle school	11.1	3.1	22.2	20.0	28.6
High school or more	8.5	3.8	0	9.1	33.3
Having disability in 2010 ^††^	**	**	**	**	**
No	11.4	4.6	13.1	15.2	28.4
Yes	64.4	33.8	47.8	74.4	81.3
Having fall history in 2010					
No	20.4	6.9	20.1	25.0	49.2
Yes	38.0	22.2	33.3	14.3	47.5
Number of chronic disease in 2010					
None	21.2	6.9	22.0	26.7	50.9
One or more	21.2	8.6	16.1	17.3	41.9
Cognitive function in 2010 ^††^					
Normal (K-MMSE ≥ 24)	9.4 **	6.2	2.9	9.6	32.1
Mild impairment (18 ≤ K-MMSE ≤ 23)	15.4	4.3	13.8	20.8	36.5
Severe impairment (K-MMSE ≤ 17)	38.1	12.5	35.7	44.6	61.8
Visual function in 2010 ^†^	**			*	
Good	16.7	7.2	16.2	17.4	49.4
Bad	28.1	6.9	26.0	35.4	48.7
Auditory function in 2010		*			**
Good	17.4	6.2	17.9	21.8	43.4
Bad	43.4	18.5	32.6	39.0	65.6
Fear-induce activity avoidance ^††^	**			*	*
No	11.3	6.5	19.6	13.0	34.8
Yes	34.1	9.4	25.0	29.3	52.4
Frequency of meeting friends	**		*	*	**
Less than several times a year or none	41.4	13.0	36.4	40.5	66.3
Once or twice a month	21.2	6.7	15.8	39.1	57.6
Everyday	16.5	6.4	16.9	19.1	40.3
Depressive symptoms ^††^	**		*	*	**
No	12.6	6.2	14.7	15.9	33.3
Yes (CES-D10 score ≥ 10)	34.6	9.7	30.1	34.2	59.6

** *p* < 0.001, * *p* < 0.01 for difference among different levels of each variable; ^†^ *p* < 0.01, ^††^ *p* < 0.001 for difference among four groups by different fear of falling status.

**Table 3:** Odds ratio (OR) ^a^ and 95% confidence interval (CI) for functional decline among Korean women aged 70 years or older.

	Model 1	Model 2	Model 3	Model 4	Model 5
	OR (95% CI)	OR (95% CI)	OR (95% CI)	OR (95% CI)	OR (95% CI)
Fear of falling (FOF)					
No FOF in both 2010 and 2012	1	1	1	1	1
Having FOF in 2010 and no FOF in 2012	1.90 (1.14–3.19) **	1.82 (1.08–3.05) *	1.85 (1.10–3.10) *	1.73 (1.02–2.91) *	1.60 (0.95–2.72)
No FOF in 2010 and having FOF in 2012	3.74 (2.36–5.94) ***	2.69 (1.63–4.43) ***	3.70 (2.33–5.89) ***	3.20 (1.99–5.11) ***	2.26 (1.36–3.75) **
Having FOF in both 2010 and 2012	6.01 (3.97–9.11) ***	4.20 (2.66–6.63) ***	5.90 (3.89–8.94) ***	4.97 (3.25–7.60) ***	3.43 (2.15–5.47) ***
Fear-induced activity avoidance					
No		1			1
Yes		1.94 (1.34–2.80) **			1.97 (1.35–2.88) **
Frequency of meeting friends					
Everyday			1		1
Once or twice a month			1.51 (0.90–2.53)		1.55 (0.92–2.62)
Less than several times a year or none			1.53 (1.04–2.26) **		1.51 (1.02–2.14) *
Depressive symptoms					
No				1	1
Yes (CES-D10 score ≥ 10)				2.26 (1.62–3.14) ***	2.15 (1.54–3.00) ***
Adjusted *R*^2^	0.472	0.481	0.476	0.489	0.500
Hosmer & Lemeshow (*p*-value)	0.929	0.296	0.987	0.255	0.326

^a^ Adjusted for age, education, marital status and living arrangement, fall history, number of chronic disease, cognitive function, visual and auditory function and disability in 2010; * *p* < 0.05, ** *p* < 0.01, *** *p* < 0.001.
